# Ultra-Processed Food and Gut Microbiota: Do Additives Affect Eubiosis? A Narrative Review

**DOI:** 10.3390/nu17010002

**Published:** 2024-12-24

**Authors:** Antonio Bevilacqua, Barbara Speranza, Angela Racioppo, Antonella Santillo, Marzia Albenzio, Antonio Derossi, Rossella Caporizzi, Matteo Francavilla, Deborah Racca, Zina Flagella, Michele Andrea De Santis, Antonio Elia, Giulia Conversa, Luciana Luchetti, Milena Sinigaglia, Maria Rosaria Corbo

**Affiliations:** Department of Agriculture, Food, Natural Resources and Engineering (DAFNE), University of Foggia, 71122 Foggia, Italy; barbara.speranza@unifg.it (B.S.); angela.racioppo@unifg.it (A.R.); antonella.santillo@unifg.it (A.S.); marzia.albenzio@unifg.it (M.A.); antonio.derossi@unifg.it (A.D.); rossella.caporizzi@unifg.it (R.C.); matteo.francavilla@unifg.it (M.F.); deborah.racca@unifg.it (D.R.); zina.flagella@unifg.it (Z.F.); michele.desantis@unifg.it (M.A.D.S.); antonio.elia@unifg.it (A.E.); giulia.conversa@unifg.it (G.C.); luciana.luchetti@unifg.it (L.L.); milena.sinigaglia@unifg.it (M.S.); mariarosaria.corbo@unifg.it (M.R.C.)

**Keywords:** microbiota equilibrium, additives, health, disease, food processing

## Abstract

The gut microbiota plays a key role in health and disease, but it could be affected by various factors (diet, lifestyle, environment, genetics, etc.). Focusing on diet, while the role of the different styles and choices (Mediterranean vs. Western diet, vegan or vegetarian diets) has been extensively studied, there are a few comprehensive papers on the effects of additives and food processing. Therefore, the main goal of this manuscript is to propose an overview of the link between ultra-processed foods and the gut microbiota based on papers and data available in the literature. The literature search was performed on PubMed and Clinicaltrials.gov, and after the selection of the most relevant articles, the paper proposes a synopsis of the effects of some classes of additives (sweeteners, preservatives, emulsifiers, glutamate, etc.), as well as of some treatments, on the gut microbiota and some pathological conditions.

## 1. Introduction

It is not easy to correctly define ultra-processed foods (UPFs); there are different criteria and classifications. However, the most widely used and accepted approach is that by NOVA [1], which classifies all food products in four clusters/categories (unprocessed or minimally processed foods, processed culinary ingredients, processed foods, and ultra-processed foods). According to this classification, UPFs are based on industrial formulations primarily composed of chemically modified substances extracted from foods, along with additives useful to promote the sensory scores and the shelf life [2]. They include carbonated soft drinks, snacks, confectionery products, ice cream and pastries, pre-prepared pasta and pizza dishes, poultry or fish nuggets and similar products (sausages, burgers, etc.), instant soups, and many others [2].

**Figure 1:**
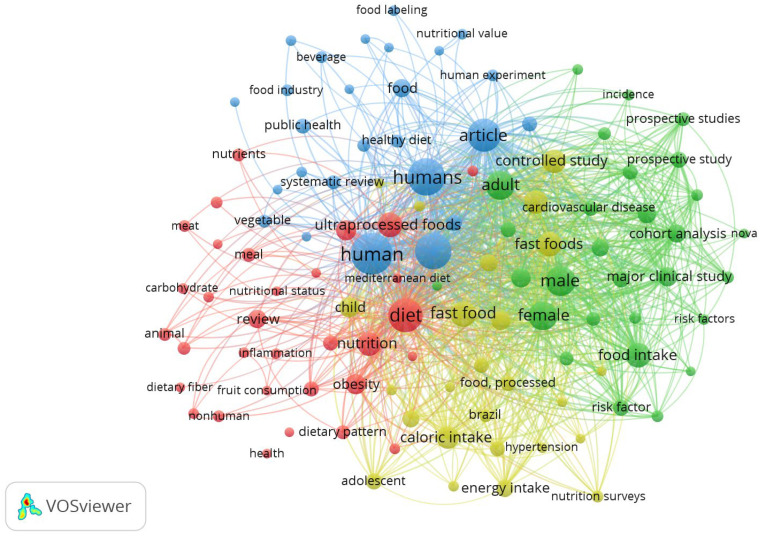
VOSviewer analysis on the keywords of the articles retrieved on Scopus for 2023 and 2024 (search performed for UPF and health).

The importance of UPFs is increasing as they represent half of the caloric daily intake in many countries, and their effect on health has been widely studied [3]; in fact, a search performed on Scopus in October 2024 revealed the existence of more than 100 research papers or reviews published in 2023 and 2024, mostly in the US, Brazil, and Europe, involving many categories (Medicine, Nursing, and Agriculture, among others), while the analysis of the records through the VOSviewer software (version 1.6.20) reveals the existence of 112 keywords recurring at least five times, and clustered in four main groups (Figure 1).

The groups are connected to four topics (UPF and nutrition, red cluster; UPF, labelling, and marketing, blue cluster; UPF and clinical trials, green cluster; UPF, health, and processing, yellow cluster). Among health issues, one of the most represented topics is that referring to the gut microbiota.

The term “microbiota” refers to the complex community of microorganisms that inhabit a defined environment—in this case, the human intestinal tract. The intestinal microbiota is composed of trillions of microorganisms, including bacteria, viruses, fungi, and protozoa, which coexist in the digestive system [4]. This microbial community not only contributes to food digestion but also plays a crucial role in the production of essential nutrients such as K and B vitamins [5].

Recent research has highlighted how the gut microbiota profoundly influences mental health and human behavior through the gut–brain axis, opening new perspectives in understanding and treating neurological and psychiatric disorders [6]. Furthermore, the gut microbiota closely interacts with the immune system, affecting the body’s immune response and protecting against pathogens [7]. The dynamism of the gut microbiota is evident in its ability to adapt and respond to external stimuli (resilience), such as diet, antibiotic use, and stress, which can alter the composition and diversity of the microbiome [8]. This underscores the importance of promoting a diverse and resilient gut microbiota through dietary choices and a healthy lifestyle.

From a therapeutic perspective, research on the gut microbiota has opened new frontiers, such as fecal therapy, which utilizes microbiota transplants to restore a healthy balance in cases of severe dysbiosis [9]. This innovative approach reveals the potential of the gut microbiota in personalized medicine and the management of metabolic, autoimmune, and inflammatory diseases.

The human gut microbiota is a complex microbial ecosystem that hosts more than 1500 bacterial species of microorganisms, distributed across over 50 different phyla [4]. The composition of the gut microbiota varies significantly with age and can be influenced by numerous factors [4]. In newborns, the gut microbiota is relatively simple and diversifies as they grow, while adults host a more complex and stable microbial community. With aging, microbial diversity tends to decrease, with a reduction in anaerobic bacteria and a higher prevalence of more resistant species, which may contribute to the predisposition to certain chronic diseases associated with old age [10].

Diet is one of the most significant factors influencing the composition of the gut microbiota. Fiber-rich diets promote the growth of beneficial bacteria such as *Bifidobacterium* and *Lactobacillus*, which are crucial for carbohydrate fermentation and short-chain fatty acid (SCFA) production [11]. Conversely, diets high in saturated fats and sugars can promote the development of a microbiota associated with metabolic conditions such as obesity and type 2 diabetes [8].

Host genetics play a fundamental role in determining the composition of the gut microbiota. Recent studies have shown that genetic variations can influence which microorganisms colonize the gut and how they interact with the host, thus affecting susceptibility to intestinal and metabolic diseases [12].

The use of antibiotics is another critical factor that can significantly alter the composition of the gut microbiota. Antibiotics eliminate both pathogenic and beneficial bacteria, creating opportunities for colonization by antibiotic-resistant pathogens. This can lead to long-term imbalances in the gut microbial community, with potential implications for host health [13].

Finally, environmental factors and lifestyle choices such as smoking, physical activity, and geographical location can influence the diversity and composition of the gut microbiota. For instance, studies have shown that geographical location can determine the prevalence of specific bacterial strains in the gut microbiota, reflecting differences in diet and the surrounding environment [14].

Focusing on diet, while the role of the different styles and choices (Mediterranean vs. Western diet, vegan or vegetarian diets) has been extensively studied, there are a few papers on the effects of additives and food processing. Some research papers and reviews are available in the literature, but it is not clear how some additives or food processing technology could directly or indirectly affect gut microbiota, nor is a comprehensive overview available on this topic. Therefore, the main goal of this research is to propose an overview of the link between UPFs and gut microbiota based on papers and data available in the literature.

## 2. Research Methodology

The literature search was performed on PubMed and ClinicalTrials.gov; the selection of keywords was essential to ensure a targeted and effective search. The main keywords used were:

- “Gut microbiota and ultra-processed foods”
- “Diet and gut microbiota”
- “Food additives effects on gut microbiota”
- “Intestinal health”
- “Processed food and intestinal flora”
- “Ultra-processed foods effect”

These keywords were selected to cover the key aspects of the research topic and ensure that all relevant studies were included.

Articles were selected following a strict criterion to ensure the quality and relevance of sources:

- Relevance of Title and Abstract: Articles were initially filtered by reading the titles and abstracts. Only those relevant to the topic of gut microbiota and UPFs were included.
- Publication Year: Studies published in the last 10 years were considered to ensure the currency of the information, unless they report essential details.
- Full-Text Availability: Only articles with full-text availability were included to allow detailed evaluation of methodologies and results.

In addition, the research papers on clinical studies were further selected and evaluated based on the Ottawa Scale, using different criteria depending on the kind of trial (if cohort study or a case control study); in the case of a cohort study, the main criteria rely on selection, comparability, and exposure, while the criteria were selection, comparability, and outcome for case-control studies. In the case of review papers, the criteria for the selection were the relevance of paper selection carried out by the authors of the review itself; the data selected on review papers were further examined individually to point out if they address the main aforementioned criteria.

## 3. Microbiota and Western Diet

Generally, lifestyles and food choices strongly impact the quali-quantitative composition of gut microbiota, and there are many details on the role of a Western diet on a qualitative shift of gut microbiota towards dysbiosis. But what are the main traits of the Western diet? According to Rininella et al. [15], there are at least four overconsumed classes of nutrients/products: saturated fats, sugars, animal proteins, and processed foods, or better UPFs.

These foods/matrices are responsible for some changes—that is, a decrease in *Bifidobacterium*, *Lactobacillus*, *Prevotella*, and *Eubacterium rectale* and an increase in *Ruminococcus*, *Bilophila*, *Klebsiella*, and other pro-inflammatory genera [15].

In addition, the negative modulation of gut microbiota is linked to the phenomenon of leaky gut, which, along with dysbiosis, is responsible for inflammation and endotoxemia [16], which in turn could lead to cardiovascular diseases, osteoporosis, autoimmune diseases, or type 2 diabetes, among others [17].

In contrast with these foods, research conducted by Toribio-Mateas et al. [18] highlights the effects of plant-based meat alternatives on gut microbiota, demonstrating how the partial replacement of animal meat with plant-based products can promote positive changes in the composition of the microbiota. By analyzing a group of 20 participants, the changes in the gut microbiota of the participants were observed following the substitution of several weekly meals with plant-based products; the results were compared with a control group. Fecal samples were analyzed through 16S rRNA sequencing, and an increase in butyrate and butyrate-producing microbial taxa was observed in the intervention group compared to the control group [18].

These findings align with the evidence reported by Dahl et al. [19], who highlight how dietary choices influence the composition and metabolism of gut microbiota, specifically, modifying the amount or ratio of carbohydrates, proteins, and fats impacts the microbiota. For example, carbohydrate-rich diets support the abundance of *Bifidobacterium*, *Prevotella*, or *Ruminococcus*, which are capable of degrading carbohydrates. In contrast, fat-rich diets support the abundance of bile-resistant microorganisms such as *Bilophila* and *Bacteroides*.

Another study highlights how changing eating habits that involve consuming processed foods containing artificial ingredients, along with the worsening of other lifestyle habits like smoking and drug use, may contribute to intestinal dysbiosis and deteriorating health, as well as the development of chronic diseases [20].

## 4. Gut Microbiota and Additives

In today’s food landscape, the consumption of UPFs has significantly increased, frequently replacing traditional fresh and unprocessed food options. Recent research has highlighted the potential impact of UPFs on human intestinal health through the modulation of gut microbiota composition; thus, Cuevas-Sierra et al. [21] examined the impact of UPF consumption on the human gut microbiota composition, considering the role of sex. Involving 359 participants, the study found that high consumption of UPFs was associated with significant variations in gut bacterial composition, with differences observed between men and women.

In women who consumed more than five servings of UPFs per day, an increase was observed in *Acidaminococcus*, *Butyrivibrio*, *Gemmiger*, *Shigella*, *Anaerofilum*, *Parabacteroides*, *Bifidobacterium*, and *Actinobacteria*. Simultaneously, a decrease in *Melainabacter* and *Lachnospira* was recorded. In men who consumed more than five servings of UPFs per day, variations were observed in microorganisms such as *Granulicatella*, *Blautia*, Carnobacteriaceae, Bacteroidaceae, Peptostreptococcaceae, Bacteroidia, and Bacteroidetes, with a decrease in *Anaerostipes* and Clostridiaceae.

In the following sections, there is evidence on the effect of some representative additives on gut microbiota, mainly sweetener, emulsifiers and thickeners, colorants, glutamate, and some preservatives.

### 4.1. Artificial and Natural Sweeteners

The consumption of artificial sweeteners is widespread in the food industry, as these substances, characterized by low or zero caloric content, low cost, and a higher sweetness level than natural sugar, are increasingly used as sugar substitutes in foods and beverages such as sugar-free desserts and soft drinks [22].

Scientific interest in artificial sweeteners, such as acesulfame potassium and aspartame, has grown significantly due to their potential effects on gut microbiota and metabolic health. Studies conducted on animals and humans have produced heterogeneous results, suggesting that the impact of artificial sweeteners on gut microbiota may vary depending on several factors, such as the amount consumed, exposure duration, and individual characteristics.

For instance, a study conducted on CD-1 mice found that acesulfame potassium can affect gut microbiota composition differently depending on the sex of the animals [23]. In male mice, a significant increase in body weight and changes in the presence of specific bacterial genera linked to carbohydrate and energy metabolism were observed, while in female mice, variations in bacterial metabolites and gene expression related to lipopolysaccharide synthesis were detected. Conversely, a study conducted on mice exposed to doses similar to the acceptable daily intake for humans did not observe significant changes in gut microbiota composition [24].

Similarly, regarding aspartame, studies on rodents have shown an increase in specific microbial groups such as *Clostridium leptum* and Enterobacteriaceae associated with the intake of this sweetener, particularly in diet-induced obese rodents [25]. However, other studies have suggested that the effect of aspartame on glucose metabolism and gut microbiota may vary among individuals, with some showing improved glucose tolerance and specific modifications in their microbiota [26].

Evidence from clinical studies on humans has yielded conflicting results. While some studies found no significant differences in gut microbiota composition between consumers and non-consumers of artificial sweeteners [27], others have suggested associations between aspartame consumption and changes in bacterial diversity and gut microbiota composition [27].

The category of artificial sweeteners also includes substances such as saccharin, sucralose, and cyclamate. Regarding saccharin, a study conducted on mice showed that this sweetener can induce disruptions in gut microbiota, including the proliferation of bacteria such as Clostridiales and *Bacteroides* and the reduction of others like *Lactobacillus* and Firmicutes [26]. Additionally, higher glucose responses were observed in saccharin-exposed mice. Studies on C57BL/6J mice indicated that saccharin intake over six months can significantly alter gut microbiota composition, with changes in the presence of various bacterial genera [23]. In humans, an association between saccharin consumption and glucose intolerance has also been observed, with some individuals showing different responses to this sweetener [26].

Regarding sucralose, studies conducted on mice have shown that this sweetener can affect gut microbiota composition, with changes in the presence of various bacterial genera after six months of exposure [23]. Furthermore, sucralose has been reported to reduce butyrate levels in the intestinal lumen, with potential implications for gut and immune health [24]. Other studies have suggested that sucralose may alter gut microbiota composition in ways that could be related to conditions such as colitis [28].

It is important to note that some artificial sweeteners, such as cyclamate, have raised safety concerns, with studies suggesting potential adverse effects such as tumor formation [29]. However, it has also been noted that cyclamate may only be harmful under certain conditions and doses and that its safety has been re-evaluated over time [30].

In conclusion, the effects of artificial sweeteners on gut microbiota and metabolic health are complex and depend on several factors. Further research is needed to fully understand these mechanisms and evaluate the potential risks associated with their consumption. The effects of the main artificial sweeteners on gut microbiota are summarized in Table 1.

Among natural sweeteners, stevia is one of the most important preparations. *Bacteroides* spp. are able to hydrolyze stevioside and rebaudioside (the main components of stevia) A to steviol [31], while other genera, e.g., enterobacteria, do not possess this ability; concerning the qualitative composition of gut microbiota, there is possible evidence of the inhibition of aerobic bacteria [31]. On other natural sweeteners (glycyrrhizin, neohesperidin dihydrochalcone, thaumatin, and monellin), there are a few or no data [32], and further efforts are required to elucidate their role on gut microbiota, if positive or negative. In this context, there is the research by Kasti et al. [33], who reviewed the most important articles dealing with this topic and found possible beneficial effects on *Lactobacillus*, *Bifidobacterium*, and *Akkermansia*, and the suppression of *Escherichia coli*, Clostridiales, and *Staphylococcus aureus*.

### 4.2. Emulsifiers and Thickeners

Emulsifiers and thickeners are widely used in the food industry to improve the texture and stability of products. However, recent studies have shown that some of these additives can influence gut microbiota and cause metabolic disturbances.

One commonly used thickener is carboxymethyl cellulose (CMC), a cellulose derivative used as a stabilizer. However, studies conducted on mice have shown that CMC intake can cause significant changes in gut microbiota and promote intestinal inflammation. Swidsinski et al. [34] observed an increase in bacterial concentration in the ileum of mice treated with CMC, along with a reduction in bacterial diversity and the onset of intestinal inflammation. Other studies confirmed that CMC intake can alter gut microbiota composition and promote the formation of intestinal tumors [35]. Additionally, in vitro models have demonstrated that CMC can increase the pro-inflammatory potential of human gut microbiota, with implications for metabolic and intestinal health [36].

González-Bermúdez et al. [37] studied the effects of different thickening agents (locust bean gum, LBG; maize hydroxypropylated distarch phosphate, Mhdp; and pre-gelatinized rice starch, gRS) on the gut microbiota of infants; the experiments were performed in vitro and in model systems. LBG positively modulated the growth of *Atopobium* and Bacteroidetes, whereas Mhdp and gRS promoted the growth of *Lactobacillus* and *Bifidobacterium*.

The consumption of K-carrageenan did not induce gut inflammation but was responsible for a modulation of gut microbiota leading to mucus degradation and to a reduced production of SCFAs [38]. In addition, Bellanco et al. [39] studied the effects of carrageenan consumption on the gut microbiota and the intestinal homeostasis of young male and female mice. The results showed an oxidative injury and cytotoxicity in epithelial cells, probably related to the capacity of gut microbiota of degrading carrageenan. These papers evidence the role of this additive in promoting a negative action on gut homeostasis by a negative modulation of microbiota.

Another thickening agent is guar gum; its effect on gut microbiota was evaluated by Barber et al. [40] through a single-arm, open label, and proof-of-concept study on healthy men. The gum is well tolerated by the microbiota, but it determined a modulation both at taxonomic and functional levels. A positive modulation was found for *Parabacteroides, Ruminococcus*, *Barnesiella*, *Lachnospira*, *Akkermansia*, *Bacteroides*, *Collinsella*, *Agathobaculum*, and *Oscillibacter*, while a negative correlation was found with *Faecalibacterium*, *Alistipes*, *Fusicatenibacter*, and *Eubacterium*. Concerning the functional analysis, the results revealed an increased activity for wide metabolic pathways, including amino acid and ribonucleotide biosynthesis and carbohydrate metabolism.

In contrast with these findings, Paudel et al. [41] suggested a possible link between guar consumption and colitis induction, as they found that guar gum affected Bacteroidota and Firmicutes, and this effect was responsible of an accumulation of intermediary metabolites succinate and lactate in mice fed with gum, along with a reduction of interleukin 18, and a decrease of gut barrier function.

Polydextrose is a bulking agent, stabilizer, thickener, and humectant. It is a soluble prebiotic fiber, applied as a replacer of sugar, starch, and fat, and for calorie reduction. From a chemical point of view, it is a branched glucose oligosaccharide, and due to its biochemical structure, it cannot be digested by human enzymes and enters the colon unaffected, where it is fermented by gut microbiota [42]. Due to these and other properties, it is also used in the case of constipation [42].

Some evidence suggests a possible positive effect on the colonic microbiota, with increased levels of SCFA, modulated microbiota, mainly Verrucomicrobia and Bacteroidetes, and increased the peristalsis [42].

The modulation of microbiota due to polydextrose consumption was also reported in a cross-over clinical trial by Costabile et al. [43], who found changes in *Lactobacillus*, *Enterococcus*, and *Ruminococcus*, but no differences in the concentration of SCFA; as a result of gut microbiota modulation, a reduction in abdominal pains was detected.

A positive effect of polydextrose was also suggested in another study conducted on mice [44], as the individuals fed with this compound showed a higher relative abundance in *Allobaculum*, and *Bifidobacterium*, along with a reduction on the taxa associated to the high-fat feeding; this effect was very important, as it counteracted the negative outcome due to a Western-style diet.

Maltodextrin is another important additive, generally used as a thickener or a filler to increase the volume in processed foods [45]. Zangara et al. [46] demonstrated that it could negatively affect gut homeostasis, by altering mucus production, and thus it increased the susceptibility to colitis. These effects were probably the result of a significant reduction of SCFA production, along with a decrease of the levels of *Akkermansia* spp. and other beneficial bacteria. Other genera that seemed to be affected by maltodextrin were *Lactobacillus*, *Bifidobacterium*, and the ratio Firmicutes/Bacteroidetes [47].

Polysorbate 80 (P80) is a commonly used emulsifier in the food industry. However, studies on mice have shown that P80 intake can alter gut microbiota composition and promote intestinal inflammation and metabolic dysfunction. Chassaing et al. [48] observed that P80 consumption caused significant changes in gut microbiota composition and increased intestinal permeability, contributing to the onset of intestinal inflammation and metabolic dysfunction. In vitro models have confirmed that P80 can increase the inflammatory potential of human gut microbiota, leading to metabolic and inflammatory disorders [36].

The role of some dietary emulsifiers was also studied by Panyod et al. [49], mainly lecithin, CMC, and sucrose fatty acids; in particular, lecithin was related to a modulation in *Faecalibaculum*, *Enterorhabdus*, and *Muribaculum*, while there is evidence of a correlation of sucrose fatty acid esters group with the [*Eubacterium*] *xylanophilum* group, *Clostridium* sensu stricto 1, and *Akkermansia*. Finally, the CMC group was related to a change in *Alloprevotella*, *Acetatifactor*, Muribaculaceae, and *Blautia*. Generally, lecithin, sucrose fatty acid esters, and CMC emulsifier feeding tended to modify gut microbiota in terms of both the α- and β-diversity indices, with a negative effect on biodiversity.

On the other hand, Robert et al. [50] found controversial and different results on the microbiota of mice fed for 13 weeks high or low-fat diets containing lecithin. Lecithins did not affect adiposity or gut inflammation and improved gut microbiota diversity by increasing Lachnospiraceae, *Lactobacillus*, and Ruminococcaceae and decreasing *Blautia* genus.

The effects of emulsifiers and thickeners on gut microbiota are summarized in Table 2.

### 4.3. Preservatives

Preservatives, whether synthetic or natural, are used to prevent undesirable changes in food caused by oxidation, enzymatic activity, and microbial growth. Sulfites are commonly used to inhibit microbial growth in foods and maintain color. Studies on beneficial intestinal bacteria have shown that even at concentrations below the established acceptable daily intake (ADI), sulfites can have bacteriostatic or bactericidal effects on certain bacterial strains. However, further research on animals and humans is necessary to fully understand the specific effects of sulfites on gut microbiota [51].

The ADIs for sodium benzoate, sodium nitrite, and potassium sorbate are 5, 0.07, and 25 mg/kg of body weight, respectively [52]. In vitro studies have shown that human gut microbiota is extremely sensitive to antimicrobial food additives. Certain species, such as *Bacteroides coprocola*, associated with Crohn’s disease and ulcerative colitis, are particularly sensitive to specific food preservatives like sodium nitrite [52]. Also, nitrites and nitrates could affect other microorganisms, as shown by the in vitro study of Hrncirova et al. [52], who reported an increase of *Bacteroides thetaiotaomicron* or *Enterococcus faecalis*, which were related to inflammation and colitis.

Additionally, studies on mice colonized with human microbiota have shown that consuming common food preservatives can reduce microbial diversity and increase the presence of bacteria from the Proteobacteria phylum. It is hypothesized that human gut microbiota exposure to preservatives, even at low doses, may cause dysbiosis [53].

Nisin, a natural food preservative with excellent antibacterial activity against Gram-positive bacteria, has an ADI of 2 mg/kg of body weight. Studies conducted on rabbits and mice have shown that nisin intake can significantly reduce the abundance of certain microbial species/groups in the gut, including *Pseudomonas*, *Clostridium*, and coagulase-positive staphylococci. However, nisin appears to have a selective effect, mainly reducing Gram-positive bacteria without significantly affecting other gut microbiota components [53].

The effects of food preservatives on the gut microbiota are summarized in Table 3.

### 4.4. Colorants

An important class of food additives is represented by colorants. A category in this class is represented by azo dyes, and some of them are not permitted as food additives, as they are proven as carcinogenic (for example, dimethylaminoazobenzene; “Butter Yellow”); however, four dyes are still permitted in the European Union and in other countries, e.g., Allura Red, Sunset Yellow, Amaranth, and Tartrazine [54,55]. Several studies assessed the connection between dyes and health, as there is possible evidence of a role of these colorants with adverse behavior in children [56].

In this context, the effects of four colorants (Allura Red, Amaranth, Sunset Yellow, and Tartrazine) on seven groups of gut bacteria have been assessed; most bacteria showed an azoreductase activity, which is thought to exert possible toxic effects. Tartrazine was also studied by Wu et al. [57], who found a direct connection of this compound with dysbiosis in juvenile crucian carp fish.

Among colorants, titanium dioxide (E171) is present in many types of foods. Studies on animals have shown that titanium dioxide nanoparticles can influence gut microbiota composition and function, increasing the risk of inflammatory and cancerous diseases [58], mainly by reducing the levels of Verrucomicrobia and Bacteroidetes, and increasing the concentration of Firmicutes by 70% [59]. Although studies suggest that titanium dioxide may have a limited effect on gut microbiota diversity [60,61], with controversial effects on *Lactobacillus*, *Bifidobacterium*, *Oscillaspora*, *Parabacteroides*, and other genera, it could still disturb the balance of the intestinal environment, along with a reduction in SCFA production and increased levels of inflammatory cytokines [62].

### 4.5. Glutamate

Monosodium glutamate (MSG) is one of the most common additives in foods [63]; it consists of glutamic acid, sodium, and water [64] and is one of the most common flavor enhancers in foods, where it is generally used to increase palatability [65].

Although it is permitted by regulatory agencies all over the world within some specific limits [63], possible health issues have been posed by many authors over the last decade. In addition, possible evidence of a connection between MSG and gut microbiota have been reviewed by Ahangari et al. [63], although the data are controversial, also depending on the target, the kind of studies and the expected outcomes. For example, Jakobsson et al. [66] and Liu et al. [67] suggested a detrimental effect on *Lactobacillus* spp. and on SCFA concentrations, while *Alistipes*, a genus probably related to the inflammatory cascade in the gut, appeared enhanced [63]. Other genera positively and negatively modulated by MSG were *Faecalibacterium*, *Citrobacter*, *Ruminococcus*, *Roseburia*, *Bifidobacterium*, and *Akkermansia*, among others [68,69,70,71].

Other evidence suggests a change also in the ratio Firmicutes/Bacteroidetes, along with a general increase in inflammatory markers, and a possible connection with glucose intolerance, and obesity together with hypertrophy of adipose tissue, hyperglycemia, hyperinsulinemia, hyperleptinemia, and even decreased glucose transport in adipocytes and muscle [63]. Most studies have been performed in animal models but suggest the need for further effort to elucidate the impact of MSG on human health.

### 4.6. Other Food Additives

Essential oils extracted from spices and herbs are often used as flavoring agents and show antibacterial activity. Studies on animals have shown that adding certain essential oils to food can improve gut microbiota and enhance the growth performance of animals. Some essential oils, such as thyme and cinnamon, have been studied for their effects on gut microbiota and intestinal diseases. These oils appear capable of inhibiting the growth of pathogenic bacteria without harming beneficial gut bacteria [72].

Organic acids such as malic acid, citric acid, and acetic acid are used as acidity regulators and flavor enhancers. These acids could either regulate intestinal pH and inhibit the growth of pathogenic bacteria while favoring acidophilic beneficial bacteria in the gut microbiota or exert bactericidal and bacteriostatic effects [73]. Another important food additive of this group is benzoic acid and its salts, mainly Na-benzoate, also discussed as a preservative; they showed in some animal models negative correlations with Coriobacteriaceae, *Prevotella*, and *Coprococcus* 1 and a positive correlation with *Ruminococcus* [74], although a study on humans showed a positive effect on *Bifidobacterium* spp. [75]. A negative effect on Coriobacteriaceae was also found for K-sorbate [76].

### 4.7. Food Processing Treatments

In addition to food additives, food processing treatments can also have a significant impact on the consumer. A recent study conducted by Zhang and Li [77] examined the effects of thermal food processing on gut microbiota diversity. Using *Silurus meridionalis* catfish and C57BL/6 mice as animal models, the authors compared the gut microbiota of subjects fed non-thermally processed food (NF) and thermally processed food (TF, 100 °C for 2–3 min). The results revealed that both animal species fed TF showed a significant reduction in gut microbiota diversity compared to those fed NF. Additionally, thermal processing triggered changes in intestinal microbial communities, although the overall composition and structure were not significantly different between NF and TF.

Also, food cooking could impair gut microbiota, with boiling exerting a positive effect and a better shift of microbiota towards eubiosis than grilling or frying at least in model systems [78]. Other studies showed that the families mostly affected by cooking methods were Lachnospiraceae and Ruminococcaceae [79].

## 5. Gut Microbiota, Additives, and Chronic Diseases

The widespread consumption of UPFs rich in additives and poor in essential nutrients is significantly altering the balance of gut microbiota. This imbalance can have significant health consequences, contributing to the onset of chronic diseases, including neurodegenerative diseases. In this context, recent research by Martinez Leo and Segura Campos [80] aims to examine the link between UPF consumption, changes in gut microbiota, and the development of such diseases, due to the fact that UPF consumption increases the risk of chronic metabolic diseases by 32%, a rise that parallels the growing prevalence of these diseases over recent decades, as reported by the World Health Organization (WHO) [80].

The dysregulation of gut microbiota is an important factor associated with neurodegenerative diseases and could serve as both a therapeutic and preventive target. Martinez Leo and Segura Campos [80] explore the effects of diets high in simple sugars and fats on the production of SCFAs by the gut microbiota. SCFAs play key roles in energy metabolism and the intermediate metabolism of various organs, including the brain, and their proper production depends on the homeostasis of gut microbiota.

The research emphasizes that the Western diet, rich in UPFs, negatively affects the composition and diversity of gut microbiota, favoring a state of dysbiosis that may contribute to the development of neurological diseases. Promoting stricter food policies and raising awareness about reducing UPF consumption could be crucial in preventing neurodegenerative diseases.

This study aligns with the findings of Atzeni et al. [81], who investigated the association between UPF consumption and fecal microbiota composition in elderly individuals with overweight/obesity and metabolic syndrome. Participants were divided into tertiles based on their UPF consumption, and diversity, dissimilarity, and fecal microbiota composition were assessed.

No significant differences in microbiota diversity were found between the UPF consumption groups, suggesting that UPF intake may not affect bacterial diversity. However, differences in fecal microbiota composition were observed among the tertiles. In the tertiles with higher UPF consumption, greater amounts of bacteria associated with metabolic disorders, such as *Alloprevotella*, *Negativibacillus*, *Prevotella*, and *Sutterella*, were found. These findings indicate that UPF consumption may influence fecal microbiota composition by promoting the growth of bacteria associated with adverse metabolic conditions.

According to the authors of this article, although UPF consumption may affect fecal microbiota composition, further studies are needed to fully understand the underlying mechanisms and their impact on metabolic and cardiovascular health. These results highlight the importance of a balanced diet with reduced UPF consumption to promote gut health and prevent metabolic diseases.

Continuing the analysis of the topic, another relevant study is by Benoit Chassaing et al. [36], who address a fundamental issue regarding the impact of synthetic food emulsifiers on human gut microbiota and intestinal inflammation. Using an advanced experimental model called the Mucosal Simulator of the Human Intestinal Microbial Ecosystem (M-SHIME), the authors demonstrated how food emulsifiers polysorbate 80 (P80) and carboxymethylcellulose (CMC) directly affect the composition and gene expression of human gut microbiota, increasing its pro-inflammatory potential. This was evidenced by the increase in bioactive flagellin levels, a molecule that stimulates an inflammatory immune response. Additionally, the results showed that these changes in human gut microbiota occur independently of the presence of a living host, suggesting a direct impact of food emulsifiers on human gut microbiota. This study provides significant evidence of how commonly used food additives can negatively affect gut health by enhancing intestinal inflammation and underscores the importance of considering the direct effects on human gut microbiota when evaluating the safety of food additives.

Juul et al. [82] explained how UPFs can cause heart problems due to their composition (high amounts of saturated fats, added sugars, and salt, all known risk factors for heart disease). Furthermore, the ultra-processing can alter the physical structure of foods, making them less satiating and leading to increased overall caloric intake, which can result in overweight and obesity, both important risk factors for heart diseases. Additionally, some food additives used in UPFs may have harmful effects on heart health, for example, by raising blood cholesterol levels or causing systemic inflammation. Finally, UPFs may affect the composition and function of gut microbiota, which has been associated with cardiovascular diseases. All these factors contribute to making UPFs a potential risk for heart health.

Song et al. [83] focused on the interconnection between UPFs and the microbiota–gut–brain axis (MGB). It highlights how modern UPFs are rich in saturated and trans fats, added sugars, salt, and food additives, all of which can have severe consequences on gut and physical health. These harmful components can cause direct modifications in gut microbiota functions and metabolism, with potential effects on the nervous system. The article highlights that while several studies have examined the negative effects of high trans-fat, added sugar, and salt consumption on gut and brain functions, relatively less attention has been paid to the impact of food additives on the MGB axis. However, data from various studies suggest that food additives may be linked to metabolic diseases and inflammation, potentially altering the composition of gut microbiota and microbial metabolites, which could, in turn, affect cognition and behavior. The article stresses the need for further studies to investigate whether intestinal dysbiosis can mediate the effects of UPFs on brain diseases and behavior, emphasizing the potential negative influence of UPF consumption on brain health.

The research by Laudanno [84] highlights the pervasive role of UPFs in daily life and global dietary consumption, emphasizing their association with serious health issues such as obesity, type 2 diabetes, cardiovascular diseases, and colorectal cancer. These products, which include snacks, carbonated drinks, ready-to-eat meals, and other highly processed foods, account for a significant portion of the calories consumed daily in many nations, especially in high-income countries. However, this trend is also spreading to other regions, such as Latin America, where UPF consumption is rapidly increasing. One of the most recent studies analyzed involves data from three prospective studies conducted in the United States over nearly three decades; the results show that men with the highest UPF consumption had a 29% higher risk of developing colon cancer, regardless of body mass index. This association was not observed in women, possibly due to hormonal differences and dietary habits. The article links colon cancer with changes in gut microbiota caused by UPFs, including additives, artificial sweeteners, acrylamide produced during cooking (such as in French fries), and BPA from packaging. These elements are associated with carcinogenesis, cardiovascular diseases, type 2 diabetes, and obesity. The article stresses the importance of addressing this issue through public policies that educate about the risks of UPFs, promote healthier eating habits, and make fresh and unprocessed products more accessible. It warns that for the first time in history, future generations may have a shorter life expectancy due to the excessive consumption of such foods and their consequences on global health.

Elechi et al. [85] provided an in-depth overview of the complex interactions between the evolving food system, dietary transition, the environment, and their impact on human gut microbiota, highlighting the consequences for global health. It emphasizes how changes in the food system over time have shaped gut microbiota, leading to epigenetic changes and a series of health challenges. Through a synthesis of current knowledge, the article outlines how food system transformations, including changes in food production, distribution, and consumption, have played a crucial role in the evolution of gut microbiota and how this has impacted human health, with a particular focus on non-communicable diseases such as obesity, cardiovascular diseases, and cancer.

## 6. Conclusions

UPFs have become increasingly pervasive in modern diets, but their consequences for human health extend far beyond mere taste satisfaction. Literature research conducted for this paper has identified a series of negative effects that these foods can have on gut microbiota and overall health, opening a window into a world of metabolic and physiological complications; a synopsis of the most important findings is in Table 4.

Gut microbiota, with its vast community of bacteria, fungi, and viruses plays a crucial role in maintaining intestinal homeostasis and regulating the immune system. However, evidence suggests that excessive consumption of UPFs can affect this delicate balance, leading to a disruption in microbiota composition. This imbalance can not only impair the metabolic and immune functions of the gut but also predispose individuals to various chronic diseases, including metabolic syndrome, obesity, and type 2 diabetes.

Moreover, UPFs seem to exert effects beyond the intestinal tract. Research has established links between UPF consumption and the onset of neurological conditions such as Alzheimer’s disease and Parkinson’s disease, suggesting that intestinal inflammation may play a role in the pathogenesis of these diseases. Likewise, UPFs can influence cardiovascular health by increasing the risk of hypertension, atherosclerosis, and other cardiovascular diseases, which could be related to changes in gut microbiota and systemic inflammation.

Cancer risk represents another significant concern associated with UPF consumption, with studies highlighting an increased incidence of tumors, particularly colorectal cancer. This risk could be attributed to both the changes in gut microbiota caused by UPF consumption and the carcinogenic effects of certain food additives present in these foods.

Additionally, the habitual consumption of UPFs can lead to long-term health problems, reducing life expectancy and increasing the prevalence of chronic diseases globally. This is particularly concerning given that these foods have become an integral part of many people’s diets worldwide.

In summary, the problem of UPFs goes beyond the simple issue of an unbalanced diet. These foods pose a significant threat to health, with long-term implications for gut microbiota and metabolic health. Addressing this problem requires a multifaceted approach that goes beyond mere individual dietary changes, involving public policies, food education, and ongoing research to fully understand the underlying mechanisms. Only through global and coordinated efforts can we hope to protect health and that of future generations from this growing threat to global well-being.

## Figures and Tables

**Table 1:** Effects of artificial sweeteners on gut microbiota.

Sweetener	Effects on Gut Microbiota	References
Acesulfame Potassium	Significant increase in body weight in male mice, changes in bacterial composition with an increase in specific genera (*Bacteroides*, *Sutterella*, *Anaerostipes*) in male mice, variations in bacterial metabolites and gene expression in female mice	[25]
Aspartame	Increase in *Clostridium leptum* and Enterobacteriaceae in mice, changes in bacterial composition in diet-induced obese mice, variations in glucose response in mice and humans	[27]
Saccharin	Proliferation of bacteria like Clostridiales and Bacteroidetes, reduction in *Lactobacillus* and Firmicutes, increased glucose response, changes in bacterial composition in mice and humans	[23,26]
Sucralose	Changes in bacterial composition with an increase in specific genera, reduction of butyrate levels in the intestinal lumen, potential correlation with conditions such as colitis	[23,24,28]

**Table 2:** Effects of emulsifiers and thickeners on gut microbiota.

Thickener/Emulsifier	Main Effects on Gut Microbiota	References
Carboxymethyl cellulose (CMC)	Increased bacterial proliferation in the intestine, induction of intestinal inflammation, changes in microbiota composition and functionality	[34,35]
Carrageenan	Mucus degradation Oxidative stress	[38,39]
Guar gum	Modulation of gut microbiota with changes in *Parabacteroides*, *Ruminococcus*, *Faecalibacterium*, *Alistipes*, *Fusicatenibacter*, and *Eubacterium*, among others Possible induction of colitis	[40] [41]
Polydextrose	Increased levels of SCFA; positive effects on peristalsis	[42]
Maltodextrin	Possible detrimental effects on *Akkermansia*, *Lactobacillus*, and *Bifidobacterium*	[46]
Polysorbate 80 (P80)	Altered microbiota composition, potential induction of intestinal inflammation, changes in cell proliferation and apoptosis	[36,48]
Lecithin and sucrose fatty acid esters	Changes in alpha and beta biodiversities	[49,50]

**Table 3:** Effects of food preservatives on gut microbiota.

Preservative	Main Effects on Gut Microbiota	References
Sulfur dioxide	Inhibition of beneficial bacterial growth, potential impact on gut microbiota composition	[51]
Sodium Benzoate, Potassium Sorbate	Variable sensitivity of bacterial species, possible effect on microbiota diversity and composition	[52]
Na-nitrite	Enhancement of pro-inflammatory genera	[52]
Nisin	Possible impact on microbiota diversity and composition, effects on pathogenic and beneficial bacterial growth	[53]

**Table 4:** Links between UPFs and health (elaboration of authors from the content of this paper).

Aspect	Changes Induced by UPFs	Health Consequences
Gut Microbiota Balance	Significant alteration	Onset of chronic diseases, including neurodegenerative diseases
Bacterial Composition	Increase in bacteria associated with metabolic disorders (e.g., *Alloprevotella*, *Negativibacillus*, *Prevotella*, *Sutterella*)	Risk of adverse metabolic conditions
SCFA Production	Reduction due to diets high in simple sugars and fats	Alterations in energy metabolism and intermediate metabolism with effects on the brain
Intestinal Inflammation	Increased pro-inflammatory potential caused by food additives (e.g., polysorbate 80, carboxymethylcellulose)	Enhanced intestinal inflammation
Metabolic Diseases	32% increased risk	Growth of chronic metabolic diseases prevalence
Neurodegenerative Diseases	Dysregulation of gut microbiota	Contribution to the development of Alzheimer’s, Parkinson’s, ALS, Friedreich’s ataxia
Cardiovascular Health	Harmful effects from saturated fats, added sugars, salt, and food additives	Increased risk of heart disease, high cholesterol, systemic inflammation
Microbiota Composition and Diversity	Negative influence from Western diet rich in UPFs	Gut dysbiosis, contribution to neurological and metabolic diseases
Cancer Risk	29% increased risk of colon cancer in men	Carcinogenesis, obesity, type 2 diabetes, cardiovascular diseases
